# PI3K p110α Blockade Enhances Anti-Tumor Efficacy of Abemaciclib in Human Colorectal Cancer Cells

**DOI:** 10.3390/cancers12092500

**Published:** 2020-09-03

**Authors:** Hyun Jung Lee, Kui-Jin Kim, Ji Hea Sung, Milang Nam, Koung Jin Suh, Ji-Won Kim, Se Hyun Kim, Jin Won Kim, Yu Jung Kim, Keun-Wook Lee, Jong Seok Lee, Jee Hyun Kim

**Affiliations:** 1 Department of Internal Medicine, Seoul National University College of Medicine, Seoul 03080, Korea; hyunjunglee@med.dongguk.ac.kr (H.J.L.); imdoctor@snu.ac.kr (K.-W.L.); jslee@snubh.org (J.S.L.); 2 Department of Internal Medicine, Dongguk University Ilsan Hospital, Goyang 10326, Korea; 3 Division of Hematology and Medical Oncology, Department of Internal Medicine, Seoul National University Bundang Hospital, Seoul National University College of Medicine, Seongnam 13620, Korea; kjkim@snubh.org (K.-J.K.); R0907@snubh.org (J.H.S.); R2786@snubh.org (M.N.); skjmd0919@snubh.org (K.J.S.); jiwonkim@snubh.org (J.-W.K.); sehyunkim@snubh.org (S.H.K.); jwkim@snubh.org (J.W.K.); cong1005@snubh.org (Y.J.K.); 4 Biomedical Research Institute, Seoul National University Bundang Hospital, Seongnam 13620, Korea

**Keywords:** colorectal cancer, abemaciclib, BYL719 (Alpelisib), cell cycle inhibition, anti-proliferative effect, migration inhibition, apoptosis, *PIK3CA* mutation

## Abstract

**Simple Summary:**

Colorectal cancer (CRC) is the third most common cancer and the second highest cause of cancer related mortality worldwide. Especially, the survival of advanced CRC patients who were failed to achieve durable remission after the anti-angiogenic and anti-epithelial growth factor receptor agents are still poor. The aim of our study was to investigate the anti-tumor activity of the CDK4/6 inhibitor, abemaciclib, as a single agent and to identify an optimal combination agent with abemaciclib in CRC cell lines. We confirmed that abemaciclib monotherapy showed anti-tumor activity and combination therapy with abemaciclib and BYL719 demonstrated synergistic effects in CRC cell lines. Moreover, our study suggested that *PIK3CA* mutation could be a predictive marker for efficacy of abemaciclib and BYL719 combination therapy. These findings provide novel insight into a possible therapeutic strategy for patients with relapsed and refractory CRC.

**Abstract:**

Targeting cell cycle regulation in colorectal cancer has not been fully evaluated. We investigated the efficacy of the CDK4/6 inhibitor, abemaciclib, and confirmed a synergistic interaction for PI3K p110α and CDK dual inhibition in colorectal cancer cell lines. Caco-2 and SNU-C4 cell lines were selected to explore the mechanism of action for and resistance to abemaciclib. In vitro and in vivo models were used to validate the anti-tumor activity of abemaciclib monotherapy and BYL719 combination therapy. Abemaciclib monotherapy inhibited cell cycle progression and proliferation in Caco-2 and SNU-C4 cells. CDK2-mediated Rb phosphorylation and AKT phosphorylation appeared to be potential resistance mechanisms to abemaciclib monotherapy. Abemaciclib/BYL719 combination therapy demonstrated synergistic effects regardless of *PIK3CA* mutation status but showed greater efficacy in the *PIK3CA* mutated SNU-C4 cell line. Growth inhibition, cell cycle arrest, and migration inhibition were confirmed as mechanisms of action for this combination. In an SNU-C4 mouse xenograft model, abemaciclib/BYL719 combination resulted in tumor growth inhibition and apoptosis with tolerable toxicity. Dual blockade of PI3K p110α and CDK4/6 showed synergistic anti-tumor effects in vivo and in vitro in human colorectal cancer cell lines. This combination could be a promising candidate for the treatment of patients with advanced colorectal cancer.

## 1. Introduction

The overall survival (OS) of colorectal cancer (CRC) has improved with the introduction of anti-angiogenic and anti-epithelial growth factor receptor (*EGFR*) agents since the early 2000s. However, CRC survival in Korea has plateaued (around 75%) over the past decade [1]. CRC is the third most common in terms of incidence and the second highest in terms of cancer related mortality after lung cancer worldwide [2]. According to the US National Cancer Institute Surveillance, Epidemiology, and End Results (SEER) reports, the five-year survival of CRC patients with distant metastases is still poor (<15%), whereas the five-year survival of locoregional disease is approximately 70–90% [3]. This reveals the unmet need of developing effective therapeutic approaches in patients with metastatic CRC. 

Cell cycle progression from G_1_ to S phase is regulated by cyclin dependent kinase (CDK) 4/6 and cyclin D1 complex-mediated phosphorylation of the retinoblastoma (Rb) tumor suppressor. The intrinsic CDK4/6 inhibitor, p16INK4a, inhibits the enzymatic activity of the CDK4/6-cyclin D1 complex. In cancer cells, the cell cycle is dysregulated by cyclin D1 overexpression, p16 loss, CDK4 mutation, and Rb loss [4]. Similarly, cell cycle dysregulation in CRC is associated with cyclin D1 dysregulation, and a variable frequency of cyclin D1 dysregulation in CRC has been reported according to the types of structural and genetic variants: *CCDN1* amplification (2.5%) [5], cyclin D1 overexpression (55%) [6], and genomic aberrations called D-cyclin-activating features (DCAFs, <10 %) [7]. The expression of cyclin D1 is regulated by several extracellular signaling pathways [8]. In particular, cyclin D levels and CDK4/6 activity are regulated by mitogenic signaling pathways. The mitogen-activated protein kinase (MAPK) pathway promotes cyclin D1 upregulation [9]. MAPK pathway genes *KRAS*, *NRAS*, and *BRAF* represent important molecular targets in colorectal cancer and serve as predictive factors in the identification of patients who potentially benefit from anti-EGFR treatment [10]. In general, mitogenic signaling via the phosphatidylinositol-3-kinase (PI3K)-AKT pathway promotes cell proliferation and tumor growth. PI3K—encoded by the *PI3KCA* gene—is activated by different receptor tyrosine kinases (such as IGFR, EGFR, VEGFR, FGFR, and RET) and activates AKT, which leads to inhibition of tuberous sclerosis complex 1/2 (TSC1/2) and consequently to activation of mTORC1/p70S6K [11]. Mitogenic signaling via the phosphatidylinositol-3-kinase (PI3K)-AKT pathway also increases cyclin D1 levels by blocking glycogen synthase kinase-3β (GSK-3β)-mediated cyclin D1 proteolysis and subcellular localization [12]. In contrast, the CDK4/6-cyclin D1 complex stimulates mammalian target of rapamycin complex 1 (mTORC1), which is located downstream of PI3K [13]. These findings give rationale for the combination of CDK4/6 inhibitors and mitogenic signaling inhibitors in CRC treatment. 

Currently, CDK is known as a modifiable key factor of cell cycle transition, and some CDK4/6 inhibitors are used in numerous clinical settings. Abemaciclib is the most recently developed selective CDK4/6 inhibitor with distinct characteristics from other selective CDK4/6 inhibitors, such as palbociclib and ribociclib. Abemaciclib has shown superior single-agent activity when compared with palbociclib and ribociclib [14,15,16], and it is more selective against CDK4 than CDK6 compared with other CDK4/6 inhibitors [17]. Consequently, abemaciclib has shown higher clinical activity by reducing the episodes of severe neutropenia that result from CDK6 inhibition [18]. Less frequent neutropenia allows continuous dosing of abemaciclib to achieve durable cell cycle inhibition, and continuous exposure to higher plasma concentrations of abemaciclib is a key mechanism for inducing apoptosis in preclinical models [19]. 

This study was designed to investigate the anti-tumor activity of the CDK4/6 inhibitor, abemaciclib, as a single agent and identify an optimal combination agent with abemaciclib in CRC cell lines. In addition, this study was performed to explore mechanisms of resistance to abemaciclib and mechanisms of action for combination therapy with abemaciclib in CRC cell lines.

## 2. Results

### 2.1. Abemaciclib Differentially Regulates Cell Proliferation Depending on Cyclin D1 and p16 Expression in Colon Cancer Cell Lines

**Figure 1:**
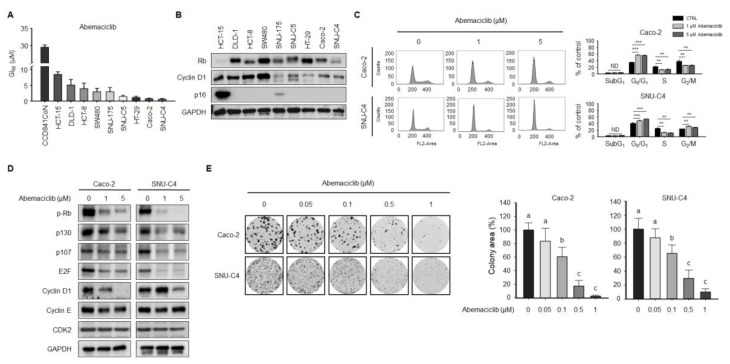
Effect of abemaciclib on cell viability and cell cycle in colon cancer cell lines. (**A**) Cells were exposed to abemaciclib at the indicated concentrations for five days. GI_50_ concentrations were calculated using CalcuSyn software. (**B**) The protein expression levels of Rb, cyclin D1, p16, and GAPDH were evaluated in HCT-15, DLD-1, HCT-8, SW480, SNU-176, SNU-C5, HT-29, Caco-2, and SNU-C4 cells. GAPDH was used as a protein-loading control (Figure S1). (**C**) Cell cycle analysis was conducted by FACScalibur after propidium iodide (PI) staining. A total of 2 × 10^6^ cells was seeded into 100-mm plates and treated with or without abemaciclib. Data are presented as histograms (black, 0 μM abemaciclib; gray, 1 μM abemaciclib; dark gray, 5 μM abemaciclib.** *p* < 0.01; and *** *p* < 0.001. (**D**) Expression levels of p-Rb, p130, p107, E2F, cyclin D1, cyclin E, and CDK2 were determined by western blot. GAPDH was used as a protein-loading control (Figure S1). (**E**) Colony-forming assays were conducted in Caco-2 and SNU-C4 cells. A total of 5 × 10^3^ cells was seeded into 6-well plates and treated with abemaciclib for seven days. Colony area was quantified using ImageJ software (National Institutes of Health, Bethesda, MD, USA). Data are expressed as the mean ± S.D. Different letters (a, b, c) indicate significant differences (*p* < 0.05).

We examined the anti-proliferative activity of abemaciclib in human normal colon epithelial CCD841CoN and CRC cell lines according to sensitivity. As shown in Figure 1A, the anti-proliferative effect of abemaciclib was relatively higher in SNU-C4, Caco-2, HT-29, and SNU-C5 cell lines (GI_50_ ≤ 2.0 μM) compared with the SNU-175, SW480, HCT-8, DLD-1, and HCT-15 cell lines (GI_50_ > 2.0 μM), whereas abemaciclib was approximately three to fifteen-fold less toxic against normal colon epithelial CCD841CoN cell compared with CRC cell lines.

Genetic alterations with potential as predictive markers for response to abemaciclib were obtained from the Cancer Cell Line Encyclopedia (CCLE) database [20]. Key genetic alterations in the nine CRC cell lines are listed in Table S1. Among the abemaciclib-sensitive CRC cell lines, all four cell lines lacked mutations in *CCND1*, *RB1*, and *KRAS*. The *CDKN2A* gene was wild-type in HT-29, Caco-2, and SNU-C4 cells, but not in SNU-C5 cells, which harbor a *CDKN2A*-silencing mutation. HT-29 and SNU-C4 cells were confirmed to have *BRAF* and *PIK3CA* mutations concurrently. 

Cyclin D1 expression was higher in some abemaciclib-resistant cell lines (HCT-15, DLD-1, HCT-8, and SW480). HCT-15 was the most resistant cell line, and it also showed high p16 expression and low Rb expression (Figure 1B). Abemaciclib-sensitive cell lines (SNU-C5, HT-29, Caco-2, and SNU-C4) reported some common features, including intact Rb expression and relatively low cyclin D1 and p16 expression, when compared with the resistant HCT-15 cell line. As more clinically relevant in vitro models, Caco-2 and SNU-C4 cells, which showed the lowest GI_50_ concentrations (<1 μM) in our experiment, were selected. 

Cell cycle analysis was performed by flow cytometry to confirm the effect of abemaciclib on cell cycle progression. Abemaciclib treatment for 48 h increased the fraction of Caco-2 and SNU-C4 cells in the G_0_/G_1_ phase and decreased that of cells in the S phase (Figure 1C). A cell cycle regulation-related gene expression assay revealed that Rb phosphorylation in Caco-2 and SNU-C4 cells decreased after exposure to abemaciclib. Cyclin E and CDK2 expression were not affected by abemaciclib treatment in Caco-2 and SNU-C4 cells (Figure 1D). Moreover, we conducted colony-forming assays to evaluate the long-term effects of abemaciclib on CRC cell lines. Colony formation in Caco-2 and SNU-C4 cells decreased after abemaciclib treatment in a dose-dependent manner (Figure 1E). 

### 2.2. Abemaciclib and BYL719 Combination Shows Additive Anti-Proliferative Activity in PIK3CA Mutated Cell Lines

**Figure 2:**
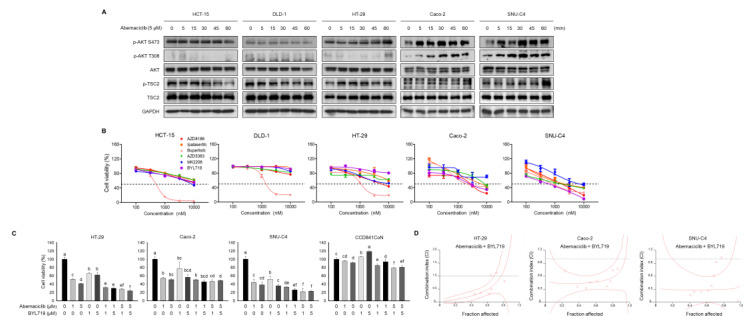
Abemaciclib and BYL719 combination enhances the anti-proliferative activity in the HCT-15, DLD-1, HT-29, Caco-2, and SNU-C4 cell lines. (**A**) HCT-15, DLD-1, HT-29, Caco-2, and SNU-C4 cells were treated with 5 μM abemaciclib for 0, 5, 15, 30, 45, and 60 min. Expression levels of p-AKT S473, p-AKT T308, total AKT, p-TSC2, and total TSC2 were determined by Western blot. GAPDH was used as a protein-loading control (Figure S2). (**B**) HCT-15, DLD-1, HT-29, Caco-2, and SNU-C4 cells were treated with the indicated concentrations of AZD8186, ipatasertib, buparlisib, AZD5363, MK2206, and BYL719 for 72 h. (**C**) HT-29, Caco-2, SNU-C4, and CCD840CoN cells were treated with 0, 1, and 5 μM abemaciclib and/or 0, 1, and 5 μM BYL719 for 72 h. Data represent the mean ± S.D. Different letters (a, b, c, d, e, f) indicate significant differences (*p* < 0.05). (**D**) HT-29, Caco-2, and SNU-C4 cells were exposed to increasing concentrations of abemaciclib and BYL719 at a fixed ratio. The anti-proliferative potential of abemaciclib combined with BYL719 was determined by calculating the combination index (CI) using CalcuSyn software according to the Chou–Talalay method.

Primary resistance cell lines against abemaciclib (HCT-15 and DLD-1) and abemaciclib sensitive cell lines (HT-29, Caco-2 and SNU-C4) were treated with 5 μM abemaciclib for 0, 5, 15, 30, 45, and 60 min. Western blot analysis of HT-29, Caco-2, and SNU-C4 cells displayed increased AKT phosphorylation by exposure to abemaciclib in a time-dependent manner (Figure 2A). Delayed TSC2 phosphorylation was also observed in HT-29, Caco-2, and SNU-C4 cells with abemaciclib treatment. However, this was not evident in HCT-15 and DLD-1 cells. To find the optimal agent for overcoming the CDK4/6 inhibitor-resistance mechanism, several PI3K-AKT inhibitors, including AZD8186, ipatasertib, buparlisib, AZD5363, MK2206, and BYL719, were tested. Dose responses for these PI3K-AKT pathway inhibitors were characterized in HCT-15, DLD-1, HT-29, Caco-2, and SNU-C4 cells (Figure 2B). BYL719 was selected as a combination agent with abemaciclib against HT-29, Caco-2, and SNU-C4 cell survival to identify the effect of selective PI3K p110α inhibition according to the *PIK3CA* mutation status of the CRC cell lines.

Abemaciclib/BYL719 combination treatment was effective in HT-29, Caco-2, and SNU-C4 cells and showed additive anti-proliferative activity in *PIK3CA* mutated HT-29 and SNU-C4 cells (Figure 2C). In addition, we observed that 5 μM of abemaciclib and 5 μM of BYL719 combination treatment showed approximately two-fold less toxic in normal colon epithelial CCD841CoN cell line compared with HT-29, Caco-2, and SNU-C4 cell lines (Figure 2C). Chou and Talalay multiple drug interaction analysis revealed further increased anti-proliferative effect of abemaciclib/BYL719 in HT-29 (CI = 0.335, Fa < 0.5), Caco-2 (CI = 0.447, Fa < 0.5), and SNU-C4 (CI = 0.226, Fa < 0.5) cells (Figure 2D and Table S2). 

### 2.3. Abemaciclib and BYL719 Combination Induces G_0_/G_1_ Phase Arrest and Alters Regulatory Protein Expression in Caco-2 and SNU-C4 Cell Lines

**Figure 3:**
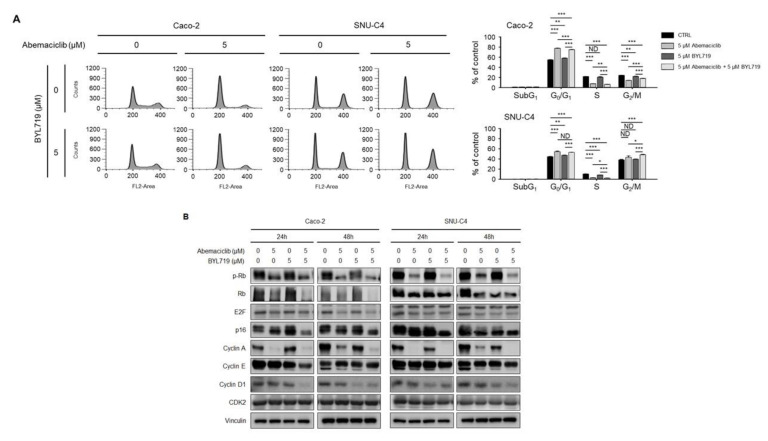
Abemaciclib and BYL719 combination inhibits the cell cycle and its regulatory proteins in Caco-2 and SNU-C4 cell lines. (**A**) Caco-2 and SNU-C4 cells were treated with 0 and 5 μM abemaciclib and/or 0 and 5 μM BYL719 for 48 h. Cell cycle distribution was analyzed by PI staining and flow cytometry. A total of 1 × 10^6^ cells was seeded in 60-mm plates and treated with the indicated concentrations of abemaciclib and BYL719 (*n* = 3). Bar graphs show the quantification of cell cycle distribution in Caco-2 and SNU-C4 cells. (Black, 0 μM abemaciclib and 0 μM BYL719; gray, 5 μM abemaciclib and 0 μM BYL719; dark gray, 0 μM abemaciclib and 5 μM BYL719; light gray, 5 μM abemaciclib and 5 μM BYL719). Data are expressed as the mean ± S.D. ND, no difference; * *p* < 0.05; ** *p* < 0.01; and *** *p* < 0.001. (**B**) The expression levels of p-Rb, Rb, E2F, p16, cyclin A, cyclin E, cyclin D1, and CDK2 were evaluated by western blot using vinculin as a protein-loading control (Figure S3).

Cell cycle analysis was performed to confirm the effects of abemaciclib and BYL719 on the cell cycle in Caco-2 and SNU-C4 cells (Figure 3A). Abemaciclib monotherapy induced G_0_/G_1_ arrest in Caco-2 and SNU-C4 cells. Abemaciclib/BYL719 combination also showed cell cycle arrest in the G_0_/G_1_ phase, but BYL719 monotherapy did not induce cell cycle arrest. 

Changes in the protein expression levels of cell cycle regulators were evaluated in Caco-2 and SNU-C4 cells according to abemaciclib and BYL719 treatment (Figure 3B). Abemaciclib monotherapy decreased the expression of phosphorylated Rb (p-Rb), Rb, E2F, and cyclin A in Caco-2 and SNU-C4 cells. Rb phosphorylation was transiently inhibited with abemaciclib treatment in Caco-2 and SNU-C4 cells, but prolonged exposure (48 h) to abemaciclib monotherapy increased Rb phosphorylation in SNU-C4 cells. BYL719 alone did not affect p-Rb expression in both cell lines. Abemaciclib/BYL719 combination decreased the protein expression of p-Rb, and this effect was sustained longer compared with abemaciclib monotherapy. Sustained expression of cyclin D1, cyclin E, and CDK2 after abemaciclib monotherapy was also observed in this study. Cyclin A, cyclin E, and cyclin D1 were downregulated with abemaciclib/BYL719 combination in Caco-2 and SNU-C4 cells. CDK2 expression was not affected by any treatment in both cell lines.

### 2.4. Abemaciclib and BYL719 Combination Inhibits Colony-Forming Activity and Cell Migration

**Figure 4:**
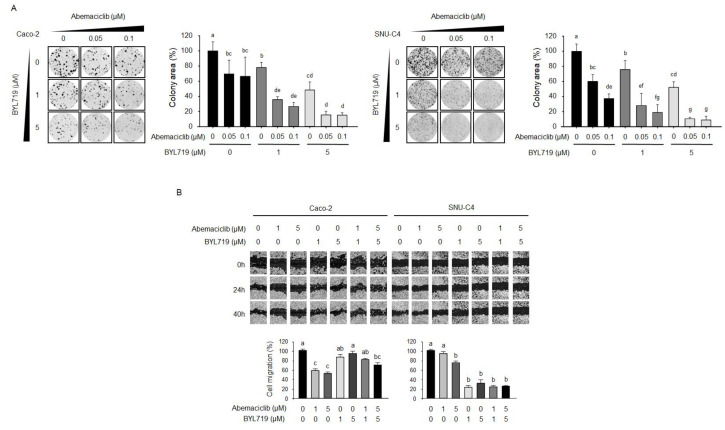
Combination effects of abemaciclib and BYL719 on colony formation and migration in Caco-2 and SNU-C4 cell lines. (**A**) Colony-forming assays were conducted in Caco-2 and SNU-C4 cells treated with 0, 0.05, and 0.1 μM abemaciclib and/or 0, 1, and 5 μM BYL719 for 10 days. Data represent the mean ± S.D. Different letters (a, b, c, d, e, f, g) indicate significant differences (*p* < 0.05). (**B**) The migration of Caco-2 and SNU-C4 cells was assessed by wound healing assays after 40 h of treatment. Representative images of the scratched areas are shown. Cell migration was quantified using ImageJ software. Different letters (a, b, c) indicate significant differences (*p* < 0.05).

We performed colony-forming assays to determine whether abemaciclib/BYL719 combination could enhance the anti-proliferative activity in Caco-2 and SNU-C4 cells compared with abemaciclib and BYL719 monotherapy (Figure 4A). In both Caco-2 and SNU-C4 cells, abemaciclib and BYL719 monotherapies significantly suppressed colony-forming activity, whereas abemaciclib/BYL719 combination synergistically increased the inhibitory effects of abemaciclib and BYL719 alone in a dose-dependent manner. 

The effect of abemaciclib/BYL719 combination on migration was evaluated using a scratch wound healing assay (Figure 4B). While abemaciclib monotherapy significantly inhibited cell migration in Caco-2 cells, BYL719 monotherapy and abemaciclib/BYL719 combination did not inhibit cell migration. In contrast, although abemaciclib alone was insufficient to alter the migration ability of SNU-C4 cells below 5 μM, abemaciclib/BYL719 combination and BYL719 monotherapy resulted in potent migration inhibition in SNU-C4 cells.

### 2.5. Abemaciclib and BYL719 Combination Inhibits Tumor Growth and Induces Apoptosis In Vivo

**Figure 5:**
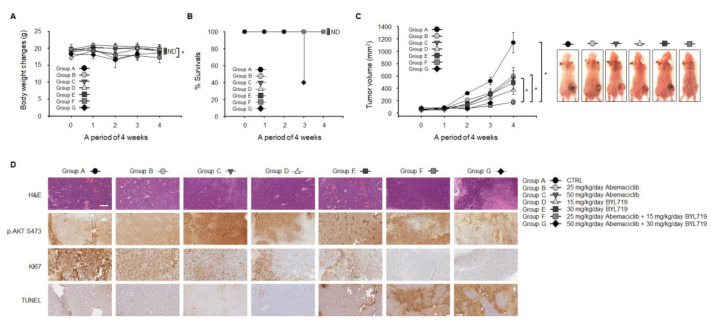
Enhanced anti-tumor efficacy of abemaciclib combined with BYL719 in colon cancer xenografts. (**A**) Athymic nude mice bearing SNU-C4 xenografts were administered with vehicle, abemaciclib (25 or 50 mg/kg/day) alone, BYL719 (15 or 30 mg/kg/day) alone, or the combination of abemaciclib and BYL719. (**B**) Body weight and survival were evaluated every seven days. (**C**) The overall anti-tumor efficacy of drugs was measured by tumor volume every seven days. Error bars represent the standard error of the mean. (**D**) Formalin-fixed paraffin-embedded tumor sections were stained with H&E and for p-AKT S473, Ki67, and TUNEL. Scale bar: 300 μm.

SNU-C4 cells showed a more potent synergistic response when treated with abemaciclib/BYL719 combination than Caco-2 cells (Figure 3A,B and Figure 4A,B). Using a mouse xenograft model of SNU-C4 cells, the in vivo anti-tumor activity of abemaciclib/BYL719 combination was evaluated. Mice were randomly assigned to receive one of the following treatments: (A) Three times weekly oral administration of vehicle, i.e., sterile water (control group); (B) daily oral administration of abemaciclib (25 mg/kg/day of body weight) in sterile water; (C) daily oral administration of abemaciclib (50 mg/kg/day of body weight) in sterile water; (D) daily oral administration of BYL719 (15 mg/kg/day of body weight) in sterile water; (E) daily oral administration of BYL719 (30 mg/kg/day of body weight) in sterile water; (F) daily oral administration of abemaciclib (25 mg/kg/day of body weight) combined with BYL719 (15 mg/kg/day of body weight) in sterile water; or (G) daily oral administration of abemaciclib (50 mg/kg/day of body weight) combined with BYL719 (30 mg/kg/day of body weight) in sterile water. During the four weeks of treatment, there was no significant change in body weight among the seven treatment groups (Figure 5A). However, three deaths were reported in group G at week 3 (Figure 5B), whereas there were no deaths in the other groups. 

The volume of tumors in the control group increased during the follow-up (Figure 5C). Abemaciclib or BYL719 monotherapy significantly inhibited tumor growth compared with the control group (*p* < 0.05). Abemaciclib/BYL719 combination showed a more potent inhibitory growth effect compared with the abemaciclib or BYL719 monotherapy groups (*p* < 0.05). 

Formalin-fixed paraffin-embedded tumor tissues from the SNU-C4 mouse xenograft model were stained with hematoxylin and eosin (H&E) and for p-AKT S473, Ki67, and TUNEL (Figure 5D). Ki67 expression dramatically decreased in the combination groups, indicating less cell proliferation, compared with the control and monotherapy groups. In the abemaciclib and BYL719 monotherapy groups, there was only a slight increase in the amount of TUNEL-positive cells, suggesting apoptosis, at high doses (30 mg/kg/day) of BYL719 monotherapy compared with the control group. However, abemaciclib/BYL719 combination treatment appeared to increase TUNEL-positive cells compared with abemaciclib or BYL719 monotherapies. 

## 3. Discussion

This study demonstrates that abemaciclib monotherapy induces cell cycle arrest and inhibits cell proliferation in CRC cell lines. Furthermore, abemaciclib has a synergistic effect in combination with BYL719 both in in vivo and in vitro CRC cell line models. This synergistic effect was more significantly demonstrated in the *PIK3CA* mutated cell lines than in the *PIK3CA* wild-type cell line. We also found that cell cycle arrest, proliferation, and migration inhibition, and apoptosis contributed to the anti-tumor activity of abemaciclib/BYL719 combination therapy in the *PIK3CA* mutated CRC cells.

Gong et al. reasoned that IC_50_ below 1 μM represent the clinically available dose with which tumors could be responsive to abemaciclib monotherapy [7]. Based on the previous literature, we decided the sensitivity cut-off of abemaciclib concentration as GI_50_ concentrations <1 μM. The limitation of our study is a concurrent use of GI_50_ and IC_50_ values in the process of confirming the effect of the drug. In the initial stage of this study, we hypothesize that the main mechanism of anti-tumor activity of abemaciclib was growth inhibition like any other previously developed CDK4/6 inhibitors. As a result, GI_50_ was used for confirming anti-tumor activity of abemaciclib in CRC cell lines. In the course of our study, we realized that abemaciclib exerts the anti-tumor activity beyond enforcing cytostatic growth arrest. Thus, we have performed cell viability test and calculate IC_50_ to analyze the anti-tumor activity of molecular-targeted agents and abemaciclib combination treatment. 

In our study, abemaciclib-sensitive cell lines presented some common features, including intact Rb expression, low p16 expression, and relatively low cyclin D1 expression. Various clinical and preclinical studies have demonstrated that Rb is the most important predictive marker for CDK4/6 inhibitors [21,22,23]. Consistent with these reports, our study showed that intact Rb is a sensitivity biomarker for CDK4/6 inhibitors. However, a recent study, which assessed abemaciclib sensitivity across many human cancer cell lines, suggested that loss of p16 and low cyclin D1 expression were associated with abemaciclib resistance [7]. Among these markers, the role of cyclin D1 and p16 expression as prognostic markers for CDK4/6 inhibitors is still controversial, even in similar groups of patients with ER+/HER2- breast cancer [24,25], and requires further evaluation in preclinical and clinical models. 

CDK2 and its regulatory cyclin-cyclin E are also known as another key factors in the progression of cell cycle transition from G_1_ to S phase as well as CDK4/6-cyclin D1 complex [26]. In addition, it was previously reported that PI3K/AKT/mTOR signaling pathway activation promotes cell cycle progression in CDK4/6 inhibitor resistant breast cancer cells through increased CDK2 and cyclin E [27]. Sustained expression of cyclin E, and CDK2 (Figure 3B) after abemaciclib monotherapy was observed in our study. This might result in delayed Rb phosphorylation in abemaciclib monotherapy. However, Rb phosphorylation and cyclin E expression decreased with abemaciclib/BYL719 combination therapy in our study. These findings suggest that CDK4/6-independent signaling pathways, which could be blocked by BYL719 combination treatment, could exist in CRC cell lines like breast cancer cell lines. In a preclinical study by Herrera-Abreu et al., elevated cyclin E expression and failed inhibition of Rb phosphorylation were detected in *PIK3CA* mutated breast cancer cell lines [28]. Prolonged CDK4/6 single inhibition and PI3K and CDK4/6 dual inhibition resulted in loss of Rb phosphorylation and reduced cyclin E expression in the same study [28]. Our study with CRC cell lines suggested that CDK2-mediated Rb phosphorylation in combination with cyclin E expression could be a mechanism of resistance to CDK4/6 inhibition.

AKT phosphorylation (Figure 2A) after abemaciclib monotherapy was also observed in this study. AKT phosphorylation is known as a potential acquired resistance mechanism for CDK4/6 inhibitors [27]. This finding indicated that activated AKT signaling by abemaciclib could be an acquired resistance mechanism against abemaciclib treatment, which could be blocked by combination therapy with PI3K-AKT signaling inhibitors. PI3K/AKT/mTOR signaling pathway activation via AKT phosphorylation is a well-known resistance mechanism against CDK4/6 inhibitors [27,28,29]. The PI3K/AKT/mTOR pathway is activated in many kinds of cancers and mainly mediated by mutations in the p110α subunit of PI3K called *PIK3CA* (>80%) [30]. *PIK3CA* mutations have been found in approximately 16–21% of CRC [31]. It was previously reported that CDK4/6 inhibitors activated the PI3K/AKT pathway through the phosphorylation of S473/T308 on AKT and CDK4/6 and PI3K inhibitor combination reduced AKT phosphorylation in *PIK3CA* mutated breast cancer cell lines. In the same study, PI3K inhibition downregulated cyclin D1 expression [24]. These findings are consistent with our findings in CRC cell lines and suggest that PI3K inhibition in combination with CDK4/6 inhibition might have the potential to overcome resistance to CDK4/6 inhibitors.

Some preclinical and early clinical studies have suggested the potential efficacy of *PIK3CA*-targeting agents [32]; however, the clinical applications of *PIK3CA* inhibitors have not advanced to late-phase clinical trials due to the lack of rationale for combination strategies and toxicities. Isoform-specific PI3K inhibitors are expected to have a wider range of therapeutic index and less off-target effects, resulting in lower toxicity. BYL719 is a selective PI3K p110α inhibitor with potent activity against its activation [33]. In our data, abemaciclib and BYL719 combination showed different outcomes according to the *PIK3CA* mutation status in each CRC cell line. SNU-C4 cells harbor the *PIK3CA* mutation, whereas Caco-2 cells do not harbor any actionable mutations in the same gene. Iida et al. reported that CDK4/6 inhibitor-resistant breast cancer cell lines were more dependent on the PI3K/AKT/mTOR pathway [34]. Almost all of the CRC cell lines used in our study were more resistant to abemaciclib (GI_50_ > 1.0 μM) compared with breast cancer cell lines (mean IC_50_ = 168 nM) [35]. CDK4/6 inhibitors limit cell proliferation by decreasing Rb phosphorylation, but inhibiting Rb phosphorylation with CDK4/6 inhibitors leads to mammalian target of rapamycin complex 2 (mTORC2)-mediated AKT activation in Rb-proficient cells [36]. Delayed AKT and TSC2 phosphorylation with abemaciclib treatment was observed in SNU-C4 cells in our study (Figure 2A). These findings support the hypothesis that there can be complex crosstalk between cell cycle and mitogenic signaling pathways in CRC cells, and that *PIK3CA* mutated CRC cells might be more dependent on the PI3K/AKT/mTOR pathway than breast cancer cells. 

Among the multiple PI3K-AKT inhibitors tested in our study, BYL719 showed potent anti-proliferative efficacy against Caco-2 and SNU-C4 cells, which was more prominent in the SNU-C4 cells. Interestingly, abemaciclib and BYL719 combination therapy significantly decreased colony-forming activity and migration in SNU-C4 cells but displayed antagonism in Caco-2 cell migration (Figure 4B). Therefore, we evaluated the in vivo efficacy of abemaciclib and BYL719 combination therapy using a mouse xenograft model of *PIK3CA* mutated SNU-C4 cells. The SNU-C4 tumor xenograft model revealed that combining abemaciclib and BYL719 could not only inhibit tumor growth (Figure 5C), but also induce apoptosis (Figure 5D). Abemaciclib has proven its cytostatic properties in various cancer types, but it sometimes exhibits cytotoxic properties alone or in combination with other agents [35,37]. In vitro data from our study gave rationale for abemaciclib and BYL719 combination therapy, and in vivo data with a *PIK3CA* mutated CRC mouse xenograft model confirmed the efficacy of abemaciclib and BYL719 combination therapy. Thus, it could be reasonable to conclude that adding BYL719 with abemaciclib leads to durable anti-tumor effects in some tumor types with *PIK3CA* mutations. 

Unfortunately, specific predictive markers for abemaciclib have yet to be discovered, except for Rb proficiency [38]. The single-agent activity of abemaciclib has resulted in limited outcomes in many clinical trials, so the focus of recent clinical trials with CDK4/6 inhibitors has moved to combination therapy and to find alternative markers in response to combination therapy. There are some ongoing clinical trials to evaluate the safety and efficacy of CDK4/6 and PI3K/AKT dual inhibition. Palbociclib with PI3K/mTOR inhibitor PF-05212384 in patients with estrogen receptor positive (ER+) metastatic breast cancer reported promising preliminary anti-tumor activity with manageable toxicities [39]. Palbociclib and PI3K inhibitor, taselisib, combination treatment in patients with *PIK3CA* mutated advanced breast cancer revealed clinical benefit with tolerable toxicities [40]. Aromatase inhibitor in combination with ribociclib and BYL719 demonstrated enhanced anti-tumor activity without evidence of drug interaction in patients with ER+/HER2- breast cancer [41]. While there is a lack of clinical data for abemaciclib because of its short developmental history, abemaciclib and selective PI3K p110α inhibitor combination are expected to be promising in clinical use. In consistent with our data, abemaciclib and selective PI3K p110α inhibitor combination was superior to single agent treatment in patient-derived xenograft models with *PIK3CA* mutated head and neck squamous cell carcinoma [42]. Until now, there are few studies to explore the efficacy and feasibility of CDK4/6 inhibitors in combination with PI3K inhibitors in patients with CRC. The findings of our study propose that *PIK3CA* mutation could be a predictive marker for the response to abemaciclib and BYL719 combination therapy in colorectal cancer treatment, and it should be validated in further preclinical and clinical trials. 

## 4. Materials and Methods 

### 4.1. Materials

Abemaciclib, BYL719, buparlisib, AZD8186, ipatasertib, AZD5363, and MK2206 were purchased from Selleckchem (Houston, TX, USA). Antibodies against the following proteins were purchased from Santa Cruz Biotechnology (Dallas, TX, USA): p107 (sc-250), p130 (sc-9963), cyclin A (sc-751), cyclin D1 (sc-753), cyclin E (sc-481), CDK2 (sc-163), and GAPDH (sc-47724). Antibodies against Rb (cs#9309), p-Rb (cs#8180), E2F (cs#3742), p16 (cs#92803), p-AKT S473 (cs#4058), p-AKT T308 (cs#9275), AKT (cs#4685), p-TSC2 (cs#3617), TSC2 (cs#4308), and vinculin (cs#13901) were purchased from Cell Signaling Technology (Danvers, MA, USA). Recombinant protein human epithelial growth factor (rhEGF) and recombinant protein human fibroblast growth factor (rhEGF) were purchased from R&D Systems (Minneapolis, MN, USA). Mitomycin C (MMC), propidium iodide (PI), RNase, and sodium dodecyl sulfate (SDS) were purchased from Sigma Aldrich (St. Louis, MO, USA). Phosphate buffered saline (PBS) and fetal bovine serum (FBS) were purchased from Gibco (Grand Island, NY, USA). Roswell Park Memorial Institute (RPMI) 1640 and Dulbecco’s modified Eagle’s medium (DMEM) medium were purchased from Welgene (Daejeon, Korea). All chemicals and reagents were of analytical grade and were obtained from commercial sources.

### 4.2. Cell Culture

HCT-15, DLD-1, HCT-8, SW480, SNU-175, SNU-C5, HT-29, Caco-2, and SNU-C4 cells were purchased from the Korean Cell Line Bank (Seoul, Korea). HCT-15, DLD-1, HCT-8, SW480, SNU-175, SNU-C5, HT-29, and SNU-C4 cells were maintained in RPMI 1640 with 10% FBS, 4mM L-glutamine, and 1% penicillin/streptomycin at 37 °C with 5% CO_2_. Caco-2 cells were maintained in DMEM with 10% FBS, 4mM L-glutamine, and 1% penicillin/streptomycin at 37 °C with 5% CO_2_. Normal colon epithelial CCD8410CoN cell line was kindly provided by Prof. Nayoung Kim at Seoul National University, Seoul, Korea. CCD841CoN cells were maintained in an Eagle’s Minimum Essential Medium (EMEM) with 10% FBS, 4mM L-glutamine, and 1% penicillin/streptomycin at 37 °C with 5% CO_2_.

### 4.3. Cell Proliferation Assay

The cell proliferation assay was performed using the CellTiter-Glo Luminescent Cell Viability Assay (Promega, Madison, WI, USA) according to manufacturer’s instructions. On day 0, 96-well plates were seeded with 3000 cells/well and incubated overnight. The next day, cells were treated with the indicated compounds. On day 4, plates were incubated for 1 h at room temperature, and 100 μL of CellTiter-Glo reagent was added to each well, followed by mixing on an orbital shaker for 5 min. Luminescence was measured on a GloMax 96-well luminometer from Promega (Madison, WI. USA).

### 4.4. Colony-Formimg Assay

Caco-2 and SNU-C4 cells were seeded into 6-well plates and grown for 72 h before being subjected to the indicated treatments for 10 days, and the media was changed at regular time intervals. After 10 days of culture at 37 °C with 5% CO_2_, colonies were washed with PBS, stained with Coomassie Brilliant Blue for 30 min at room temperature, then washed with water, and air-dried. The colonies were photographed using the ChemiDoc Touch (Bio-Rad) and measured using ImageJ software (National Institutes of Health, Bethesda, MD, USA).

### 4.5. Cell Migration

Caco-2 and SNU-C4 cells were seeded into 96-well plates and grown for 24 h. Confluent monolayers were gently scratched using a WoundMaker (Essen Bioscience, Ann Arbor, MI, USA). Cells were washed twice with PBS to remove floating cells and then incubated for 40 h in growth medium supplemented with 10 ng/mL rhEGF, 10 ng/mL rhFGF2, and 10 μg/mL mitomycin C. The rate of cell migration was expressed as the area of the scratch relative to total area of the cell-free region immediately after the scratch using IncuCyte Zoom (Essen Bioscience, Ann Arbor, MI, USA).

### 4.6. Cell Cycle Analysis

Caco-2 and SNU-C4 cells were seeded into 100-mm plates and grown overnight and were then subjected to the indicated drug treatments for 48 h. After trypsinization, cells were washed twice in PBS, fixed overnight at 4 °C in ethanol, washed three times with PBS, and incubated in PBS containing 20 μg/mL PI and 100 μg/mL RNAse at 37 °C for 30 min. After washing in PBS, cells were resuspended in 1 mL PBS and examined using a FACSCalibur flow cytometer (BD Biosciences, Franklin Lakes, NJ, USA). Cell cycle distribution was determined using FlowJo software (Tree Star, Ashland, OR, USA).

### 4.7. Combination Index Analysis

HT-29, Caco-2, and SNU-C4 cells were seeded into 96-well plates at 3000 cells/well in a total volume of 100 μL basal media containing 10% FBS. The following day, cells were treated in pentaplicate with single agents and their fixed-ratio combination for 72 h over a seven-point titration, which was centered on the single-agent concentrations that inhibited viability by 50% (IC_50_). Cell viability was measured by the CellTiter-Glo Luminescent Cell Viability Assay (Promega, Madison, WI, USA) according to manufacturer’s instructions. Combination index (CI) scores were calculated as previously described [43] using CalcuSyn software (Biosoft, Ferhuson, MO, USA). This software uses the Chou–Talalay combination index method, which is based on the median-effect equation, itself a derivation from the mass-action law. For this analysis, abemaciclib was combined with BYL719 at a constant ratio determined by GI_50_ abemaciclib/IC_50_ BYL719. We entered the resulting proliferation data, along with the data obtained from single drug treatments, into CalcuSyn to determine a CI value for each combination point, which quantitatively defines synergy (CI < 1), additivity (CI = 1), and antagonism (CI > 1). 

### 4.8. Western Blot Analysis

Colon cancer cell lines lysates were obtained by centrifugation at 12,000× *g* for 30 min at 4 °C. Protein concentration in the supernatant was measured by Bradford assay (BioLegend, San Diego, CA, USA). Proteins (20 μg) were separated by SDS polyacrylamide gel electrophoresis, transferred to a polyvinylidene difluoride membrane (Bio-Rad, Hercules, CA, USA) that was blocked in blocking buffer containing 5% skim milk, and then probed overnight with primary antibodies. Secondary antibodies conjugated with horseradish peroxidase (1:4000 dilution; Bio-Rad) were applied for 1 h. Immunoreactivity was detected by enhanced chemiluminescence (Biosesang, Seongnam, Korea) and a ChemiDoc Touch imager (Bio-Rad).

### 4.9. Mouse Xenograft 

All mice were housed in a specific pathogen-free facility at the Seoul National University Bundang Hospital, Seongnam, Korea. The project was approved by the Institutional Animal Care and Use committee of Seoul National University Bundang Hospital (IACUC approval number: 51-2018-046). For xenograft mouse studies, male athymic nude mice, weighing 26–28 g (5 weeks old), were purchased from Orient Bio Co. (Kapyong, Korea). Mice were provided with NIH-07 rodent chow (Zeigler Brothers, Gardners, PA, USA) purchased from Central Lab Animal Inc. (Seoul, Korea). Animals were acclimated to temperature (20–24 °C) and humidity (44.5–51.8%) and a 12-h light/dark cycle for one week prior to use. SNU-C4 cells (1 × 10^7^) were subcutaneously implanted with Matrigel (BD Biosciences, San Jose, CA, USA) into the flank of each mouse. Ten days after cell inoculation, when palpable tumors were observed, mice were randomly assigned to receive one of the following treatments: (A) Three times weekly oral administration of vehicle, i.e., sterile water (control group), (B) daily oral administration of abemaciclib (25 mg/kg/day of body weight) in sterile water, (C) daily oral administration of abemaciclib (50 mg/kg/day of body weight) in sterile water, (D) daily oral administration of BYL719 (15 mg/kg/day of body weight) in sterile water, (E) daily oral administration of BYL719 (30 mg/kg/day of body weight) in sterile water, (F) daily oral administration of abemaciclib (25 mg/kg/day of body weight) combined with BYL719 (15 mg/kg/day of body weight) in sterile water, or (G) daily oral administration of abemaciclib (50 mg/kg/day of body weight) combined with BYL719 (30 mg/kg/day of body weight) in sterile water. The mice (five per treatment group) were weighed, and tumor areas were measured throughout the study. Treatments were continued for four weeks, and the mice were euthanized by CO_2_ asphyxiation, weighed, and subjected to necropsy. The volume and weights of xenograft tumors were recorded. Selected tissues were further examined by routine H&E staining and immunohistochemical analyses.

### 4.10. Immunohistochemistry

Paraffin-embedded tissue blocks from the xenograft tumors were extracted and sectioned at a thickness of 5 μm. Tissue sections from mouse xenograft tumors, mounted on poly-L-lysine-coated slides, were deparaffinized by standard methods. Endogenous peroxidase was blocked by 3% hydrogen peroxide in PBS for 10 min. Antigen retrieval was done for 5 min in 10 mM sodium citrate buffer (pH 6.0) heated at 95 °C in a steamer, followed by cooling for 15 min. Slides were washed with PBS and incubated for 1 h at room temperature with a protein-blocking solution (VECTASTAIN ABC kit, Vector Laboratories, Burlingame, CA, USA). Excess blocking solution was drained, and samples were incubated overnight at 4 °C with one of the following: A 1:500 dilution of p-AKT S473 and KI67 antibodies or a 1:200 dilution of TUNEL antibody. Sections were then incubated with biotinylated secondary antibody followed by streptavidin (VECTASTAIN ABC kit). Color was developed by exposing the peroxidase to diaminobenzidine reagent (Vector Laboratories), which forms a brown reaction product. Sections were then counterstained with Gill’s hematoxylin (Sigma) for 1 min. Brown staining identified p-AKT S473, KI67, and TUNEL expression.

### 4.11. Statistical Analysis

Statistical analyses were performed using SPSS v.12.0 software (SPSS Inc., Chicago, IL, USA). One-way analysis of variance was used for comparisons among groups. Significant differences between mean values were assessed with Duncan’s test. A *p-*value < 0.05 was considered statistically significant. The Student’s t-test was also used to compare two independent groups. * *p* < 0.05; ** *p* < 0.01; or *** *p* < 0.001.

## 5. Conclusions

In the present study, our findings suggest that abemaciclib and BYL719 combination therapy is effective in preclinical CRC cell line models. Cell cycle arrest, proliferation and migration inhibition, and apoptosis are the main contributors to the anti-tumor activity of abemaciclib and BYL719 combination therapy. Moreover, our study suggests that *PIK3CA* mutation could be an additional predictive marker for the efficacy of abemaciclib in combination with BYL719. These findings provide novel insight into a possible therapeutic strategy for patients with relapsed refractory CRC.

## Supplementary Materials

The following are available online at https://www.mdpi.com/2072-6694/12/9/2500/s1, Table S1: Key genetic alterations in 9 colorectal cancer cell lines, Table S2: The synergistic activity of abemaciclib with BYL719 in Caco-2 and SNU-C4 cell lines, Figure S1: Uncropped Western blot images of indicated proteins in whole cell lysates from Caco-2 and SNU-C4 cells treated with and without abemaciclib, related to Figure 1B,D, Figure S2: Uncropped Western blot images of indicated proteins in whole cell lysates from Caco-2 and SNU-C4 cells treated with and without abemaciclib, related to Figure 2A, Figure S3: Uncropped Western blot images of indicated proteins in whole cell lysates from Caco-2 and SNU-C4 cells treated with abemaciclib and/or BYL719, related to Figure 3B.
