# Supplementary material for: PI3K p110α Blockade Enhances Anti-Tumor Efficacy of Abemaciclib in Human Colorectal Cancer Cells
Source: Cancers (Basel). 2020 Sep 3;12(9):2500. doi: 10.3390/cancers12092500 (PMC7564416; doi:10.3390/cancers12092500)
Supplement: Supplementary file 1 [file cancers-12-02500-s001.pdf]

cancers

Supplementary Information for

Article

PI3K p110α Blockade Enhances Anti-Tumor Efficacy of Abemaciclib in Human Colorectal Cancer Cells

Hyun Jung Lee [1,2,†], Kui-Jin Kim [3,4,†], Ji Hea Sung [3], Milang Nam [3], Koung Jin Suh [3], Ji-Won Kim [3], Se Hyun Kim [3], Jin Won Kim [3], Yu Jung Kim [3], Keun-Wook Lee [1,3], Jong Seok Lee [1,3] and Jee Hyun Kim [1,3,*]

[1] Department of Internal Medicine, Seoul National University College of Medicine, Seoul, 03080, Republic of Korea; hyunjunglee@med.dongguk.ac.kr (H.J.L.); imdoctor@snu.ac.kr (K.W.L.); jslee@snubh.org (J.S.L.); jhkimmd@snu.ac.kr (J.H.K)

[2] Department of Internal Medicine, Dongguk University Ilsan Hospital, Goyang, 10326, Republic of Korea

[3] Division of Hematology and Medical Oncology, Department of Internal Medicine, Seoul National University Bundang Hospital, Seoul National University College of Medicine, Seongnam, 13620, Republic of Korea; kjkim@snubh.org (K.J.K.); R0907@snubh.org (J.H.S.); R2786@snubh.org (M.L.N); sehyunkim@snubh.org (S.H.K.); skjmd0919@snubh.org (K.J.S.); jiwonkim@snubh.org (J.W.K.); jwkim@snubh.org (J.W.K.); cong1005@snubh.org (Y.J.K.); imdoctor@snu.ac.kr (K.W.L.); jslee@snubh.org (J.S.L.); jhkimmd@snu.ac.kr (J.H.K)

[4] Biomedical Research Institute, Seoul National University Bundang Hospital, Seongnam, 13620, Republic of Korea; kjkim@snubh.org (K.J.K)

* Correspondence: jhkimmd@snu.ac.kr; Tel: +82-31-787-7022 Fax: +82-31-787-4052

† These authors have contributed equally to this work.

Table S1. Key genetic alterations in 9 colorectal cancer cell lines.

	**Primary resistance cell lines to CDK4/6 inhibitors**					Sensitive cell lines to CDK4/6 inhibitors			
	**HCT-15**	**DLD-1**	**HCT-8**	**SW480**	**SNU-175**	SNU-C5	HT-29	Caco-2	SNU-C4
CCND1	WT	-	-	WT	WT	WT	WT	WT	WT
CDKN2A	WT	-	-	WT	DEL	Silent	WT	WT	WT
RB1	WT	-	-	WT	R798Q<br>D921D	WT	WT	WT	WT
KRAS	G13D	G13D	G13D	G12V	A59T	WT	WT	WT	WT
BRAF	WT	WT	WT	WT	WT	V600E	V600E<br>T119S	WT	D22N
PIK3CA	E545K<br>D549N	E545K<br>D549N	E545K<br>D549N	WT	WT	H1047R	P449T	WT	E545G<br>V71I
PETN	WT	WT	WT	WT	WT	WT	WT	WT	F288fs<br>F288S

Table S2. The synergistic activity of abemaciclib with BYL719 in HT-29, Caco-2, and, SNU-C4 cell lines.

**HT-29**					**Caco-2**					**SNU-C4**				
**Fa**	**CI**	**Est. S.D.**	**Abemaciclib**	**BYL719**	**Fa**	**CI**	**Est. S.D.**	**Abemaciclib**	**BYL719**	**Fa**	**CI**	Est. S.D.	Abemaciclib	BYL719
0.02	0.011	0.025	2×10^{-6}	10^{-5}	0.02	0.513	0.579	2×10^{-4}	9×10^{-4}	0.02	0.336	1.306	2×10^{-4}	2×10^{-4}
0.05	0.025	0.04	3×10^{-5}	2×10^{-4}	0.05	0.472	0.387	0.001	0.006	0.05	0.313	0.805	0.001	0.001
0.1	0.048	0.057	3×10^{-4}	0.001	0.1	0.447	0.271	0.005	0.026	0.1	0.299	0.549	0.005	0.005
0.15	0.072	0.068	9×10^{-4}	0.004	0.15	0.437	0.212	0.012	0.065	0.15	0.293	0.432	0.013	0.011
0.2	0.098	0.077	0.002	0.012	0.2	0.432	0.173	0.024	0.131	0.2	0.29	0.359	0.026	0.022
0.25	0.127	0.084	0.005	0.026	0.25	0.429	0.145	0.043	0.233	0.25	0.288	0.307	0.045	0.038
0.3	0.158	0.089	0.01	0.051	0.3	0.43	0.124	0.072	0.385	0.3	0.287	0.267	0.073	0.062
0.35	0.193	0.092	0.019	0.096	0.35	0.432	0.108	0.113	0.608	0.35	0.286	0.235	0.113	0.096
0.4	0.234	0.094	0.035	0.174	0.4	0.435	0.096	0.174	0.933	0.4	0.286	0.209	0.17	0.145

0.45	0.28	0.095	0.061	0.306	0.45	0.44	0.088	0.261	1.405	0.45	0.286	0.187	0.252	0.215
0.5	0.335	0.096	0.107	0.533	0.5	0.447	0.083	0.39	2.099	0.5	0.286	0.171	0.371	0.316
0.55	0.401	0.098	0.186	0.928	0.55	0.457	0.083	0.583	3.135	0.55	0.287	0.159	0.545	0.464
0.6	0.482	0.108	0.327	1.634	0.6	0.469	0.087	0.879	4.722	0.6	0.288	0.153	0.807	0.687
0.65	0.583	0.134	0.59	2.948	0.65	0.484	0.095	1.347	7.239	0.65	0.289	0.155	1.215	1.035
0.7	0.716	0.19	1.108	5.539	0.7	0.505	0.107	2.126	11.43	0.7	0.291	0.165	1.882	1.603
0.75	0.899	0.296	2.218	11.09	0.75	0.534	0.124	3.515	18.89	0.75	0.294	0.185	3.047	2.596
0.8	1.166	0.489	4.912	24.56	0.8	0.575	0.147	6.248	33.59	0.8	0.298	0.22	5.29	4.506
0.85	1.602	0.873	12.86	64.29	0.85	0.639	0.183	12.54	67.41	0.85	0.304	0.276	10.31	8.787
0.9	2.447	1.792	46.16	230.8	0.9	0.756	0.246	31.64	170	0.9	0.313	0.372	25.04	21.33
0.95	4.888	5.312	363.8	1819	0.95	1.051	0.415	141	758	0.95	0.332	0.593	104.9	89.39
0.99	23.37	51.85	34787	2E+05	0.99	2.614	1.54	3830	20584	0.99	0.395	1.572	2486	2117

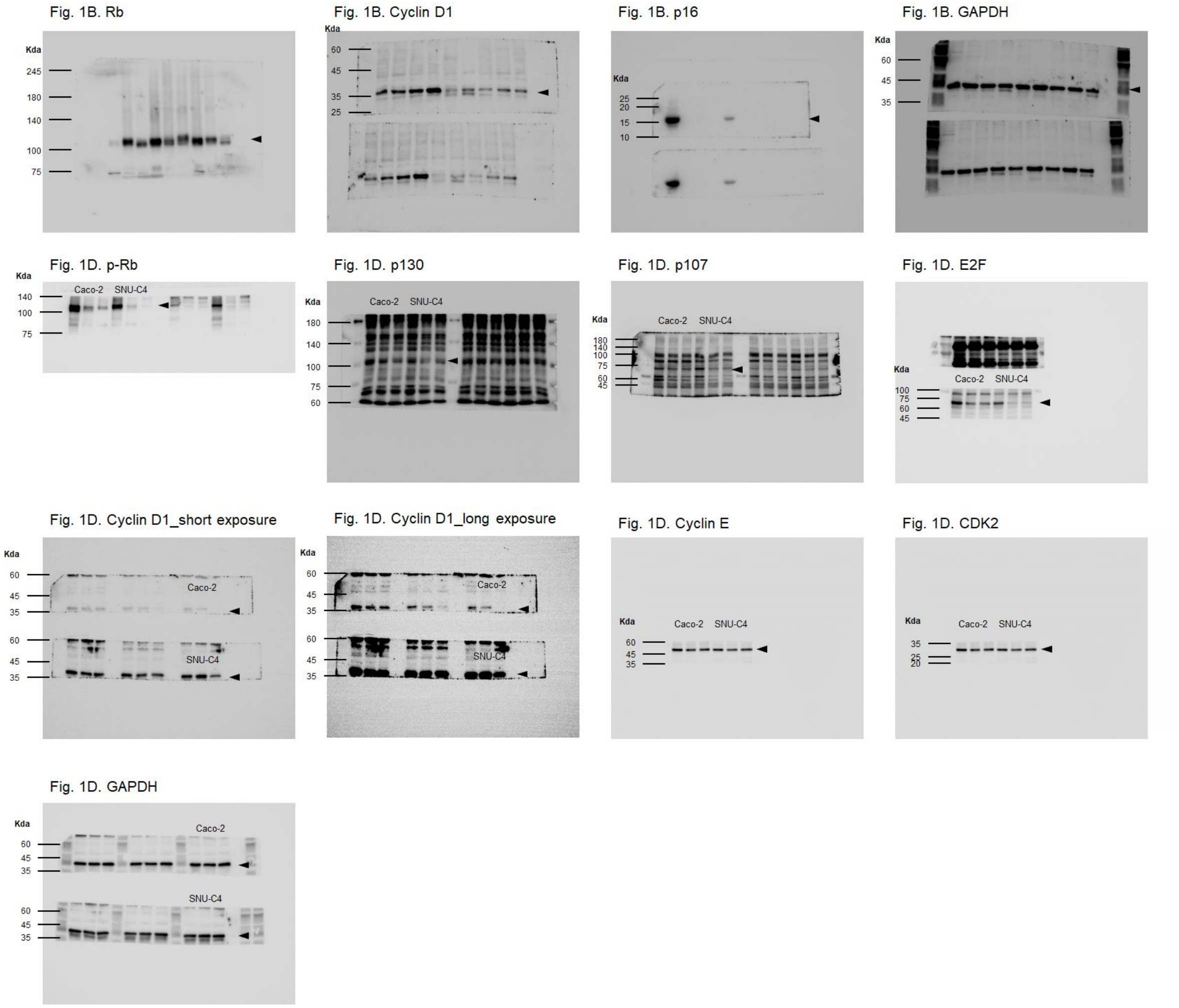

Figure S1. Uncropped Western blot images of indicated proteins in whole cell lysates from Caco-2 and SNU-C4 cells treated with and without abemaciclib, related to Figure 1B and 1D.

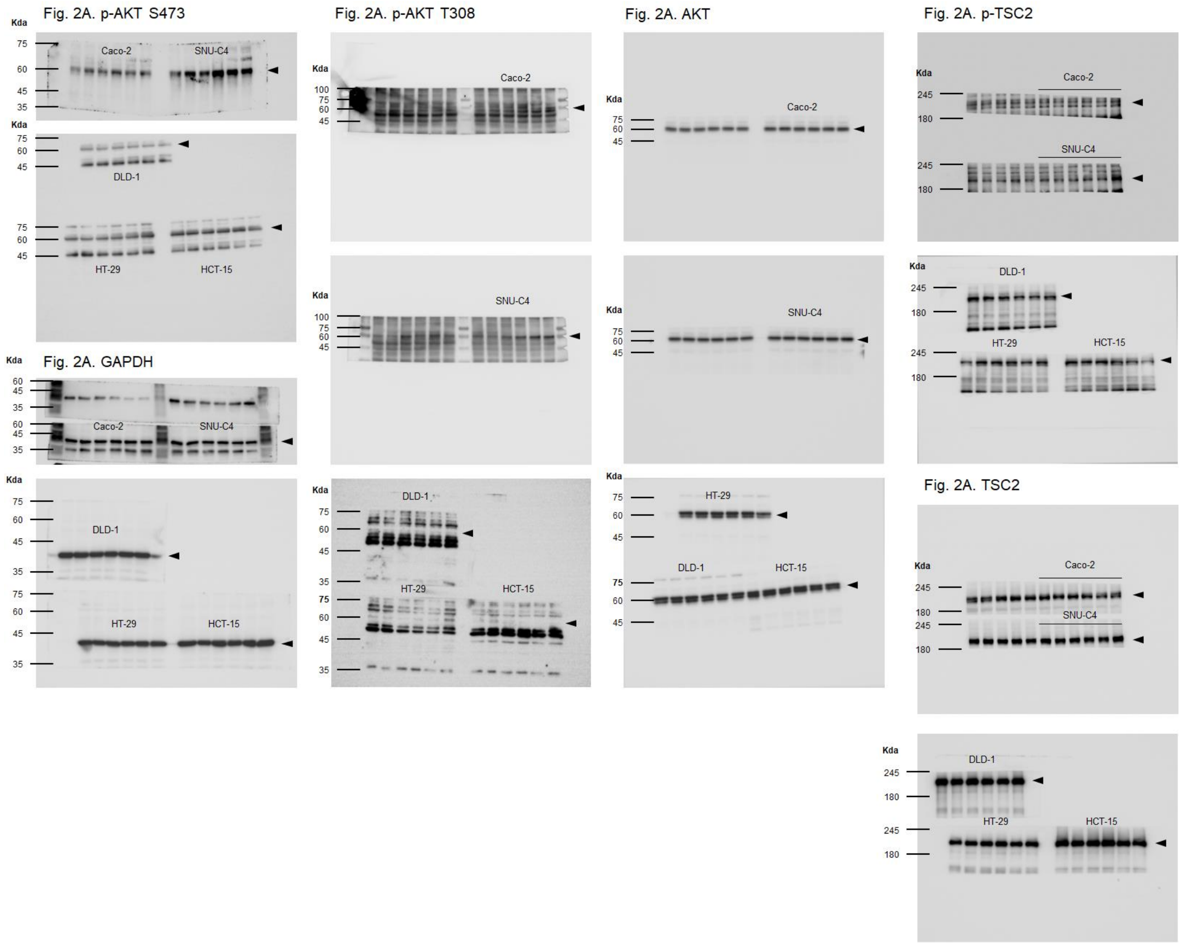

Figure S2. Uncropped Western blot images of indicated proteins in whole cell lysates from HCT-15, DLD-1, HT-29, Caco-2, and SNU-C4 cells treated with and without abemaciclib, related to Figure 2A.

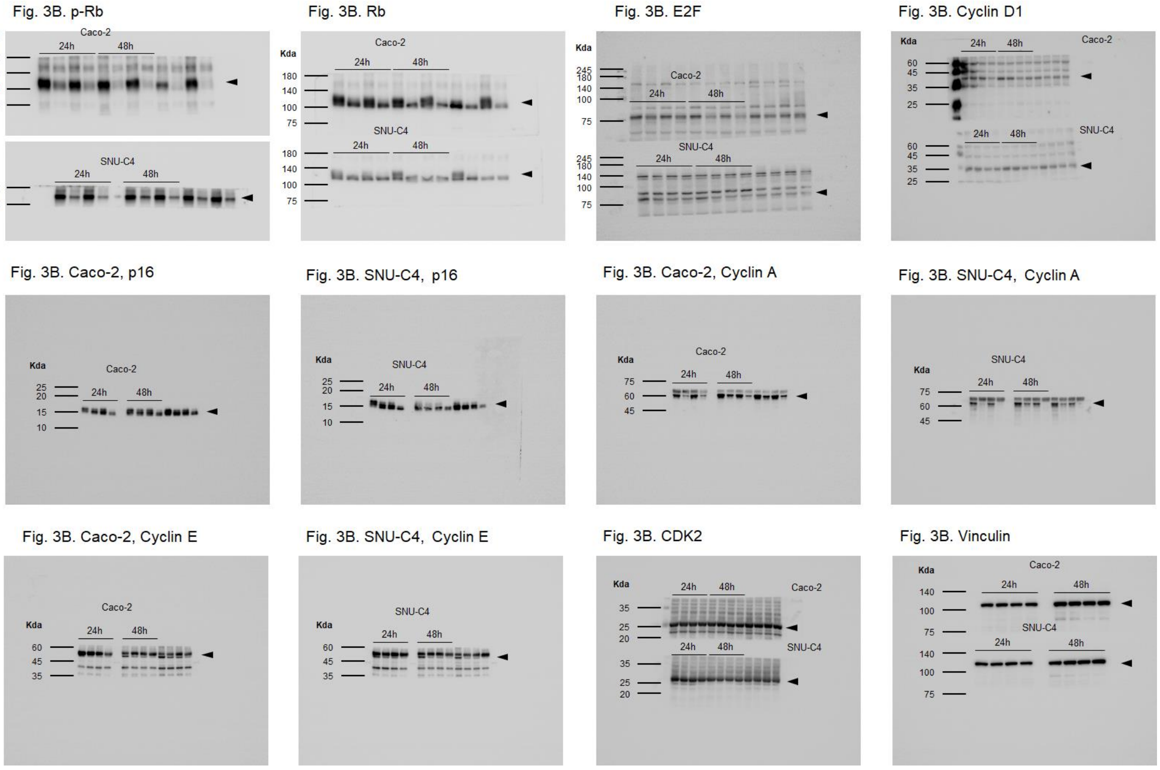

Figure 3S. Uncropped Western blot images of indicated proteins in whole cell lysates from Caco-2 and SNU-C4 cells treated with abemaciclib and/or BYL719, related to Figure 3B.
